# Supplementary figures and images for: Nodal patterning without Lefty inhibitory feedback is functional but fragile
Source: eLife. 2017 Dec 7;6:e28785. doi: 10.7554/eLife.28785 (PMC5720593; doi:10.7554/eLife.28785)

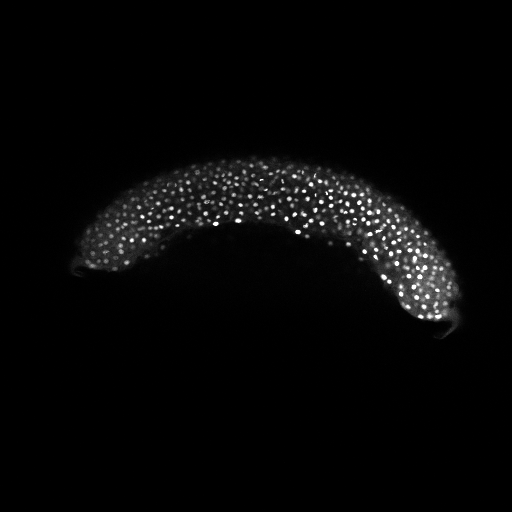

Supplement: Source code 1. [file elife-28785-code1.zip › Code Package for eLife20171129/Example Run/Sytox_lft_50_1.tif]
